# Microstructural Control of CaO–Al_2_O_3_–SiO_2_ Glass Ceramics by Oxidation and Mixing with Nucleation Agents

**DOI:** 10.1021/acsomega.2c03799

**Published:** 2022-09-08

**Authors:** Shingo Machida, Kei Maeda, Ken-ichi Katsumata, Atsuo Yasumori

**Affiliations:** Department of Material Science and Technology, Faculty of Advanced Engineering, Tokyo University of Science, 6-3-1 Niijuku, Katsushika-ku, Tokyo 125-8585, Japan

## Abstract

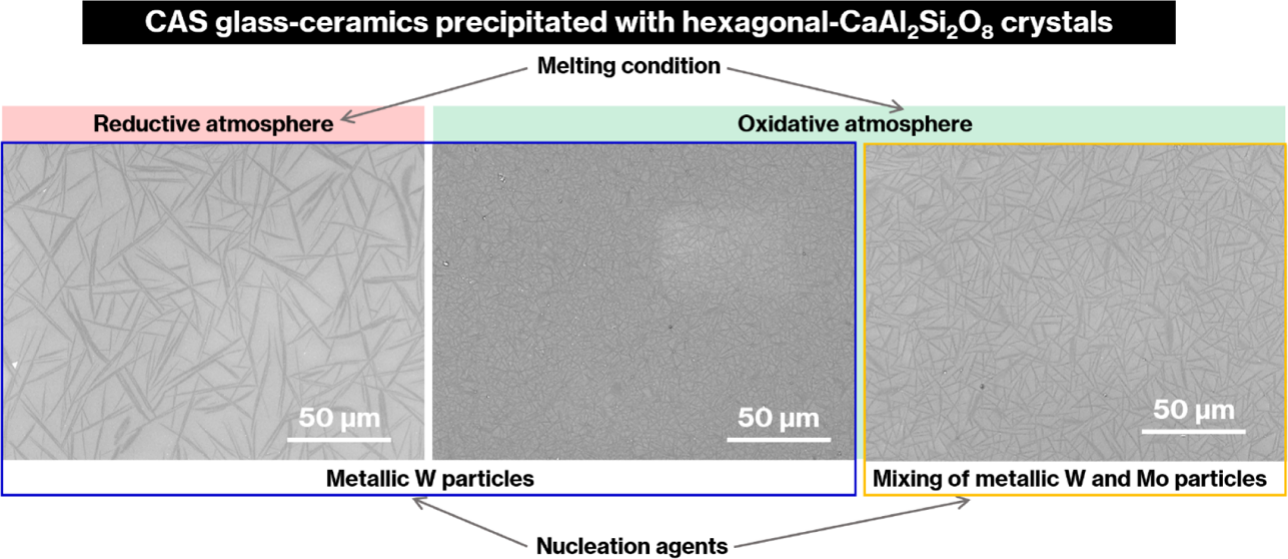

Microstructural control of CaO–Al_2_O_3_–SiO_2_ (CAS) glass ceramics (GCs) was achieved by oxidation and mixing with nucleation agents. CAS GCs were precipitated with hexagonal platy particles of metastable CaAl_2_Si_2_O_8_ layered crystals (CAS GC-H), which are typically prepared under a reductive atmosphere that forms metallic Mo or W particles as nucleation agents. The average particle size of crystals decreased significantly from 50 to 11 μm when the CAS GC-H containing metallic W particles was prepared under an oxidative atmosphere. Compared to this CAS-GC-H, the crystal particle size increased from 8–20 to 10–30 μm when the CAS GC-H was prepared by mixing glass cullet containing metallic Mo and that containing metallic W particles. These results indicate that one microstructure of CAS GC-H is controlled on the micrometer scale from a parent glass with one composition by varying the experimental conditions related to the glass melting state.

## Introduction

The discovery of glass ceramics (GCs)^1^ promoted research into their properties, which are superior to those of monolithic glass materials.^2−20^ GCs are composed of crystals such as enstatite (MgSiO_3_), fluorcanasite (K_2_Na_4_Ca_5_Si_12_O_30_F_4_),^8,9^ and lithium disilicate (Li_2_Si_2_O_5_),^10^ which improve the mechanical properties of the glass, such as an increase in the fracture toughness.^3−12^ GCs with lower fracture toughness have also attracted attention with respect to their fracture behavior. Notably, hexagonal platy particles of metastable CaAl_2_Si_2_O_8_ layered crystals in the CaO–Al_2_O_3_–SiO_2_ (CAS) system form a house-of-cards structure that exhibits crack deflection and microcracks by Vickers indentation^6,13−17^ in a manner similar to mica GCs.^6^ Microstructural control of this CAS GC by changing the chemical composition of the parent glasses has also enabled investigation of the effect of crystal size/fraction in the microstructure on the fracture toughness and indicated that a microstructure with a relatively high crystal fraction decreases the fracture toughness.^16^ However, this microstructure differs from that with a relatively low crystal fraction that has relatively high fracture toughness.^16^ In addition, there is a general relationship between the glass composition and the mechanical properties. It is thus desirable that a parent glass with one composition results in one type of microstructure with different crystal sizes. Control of the microstructure of CAS GC precipitated with hexagonal platy particles of metastable CaAl_2_Si_2_O_8_ (CAS GC-H) is a promising approach. The nucleation agents employed can thus be appropriately adjusted.

Here, we report on the microstructural control of CAS GC-H by oxidation and mixing with nucleation agents. The nucleation of CAS GC-H proceeds during the glass melting stage. Metallic Mo particles are formed via the reduction of MoO_3_ by carbon.^13−19^ When a remelting process is conducted to obtain a homogeneous glass specimen, the addition of a glass batch containing carbon is necessary to form a reductive atmosphere. However, such a glass batch should be removed to decrease the sizes of metallic Mo particles by oxidation. Metallic tungsten (W) particles obtained in a similar fashion to metallic Mo particles also act as nucleation agents for the MgO–Al_2_O_3_–SiO_2_ (MAS) system.^18^ Based on the differences in glass color from that using metallic Mo particles,^18^ the size of the W particle nucleation agents differ. Mixing of metallic Mo and W particles is thus a promising approach for control of the CAS-GC-H microstructure. Therefore, CAS GC-H specimens were prepared using an oxidative atmosphere in the remelting process or mixing of glass cullet containing metallic Mo and/or W particles.

## Experimental Section

Calcium carbonate (CaCO_3_), aluminum oxide (Al_2_O_3_), silica (SiO_2_), and tungsten oxide (WO_3_) were obtained from Wako Pure Chemical. MoO_3_ and carbon were obtained from Kojundo Chemical Laboratory. The CAS GC-H products were prepared according to a previously reported procedure.^13−17^ The melting conditions were 1550 °C for 1 h under air in an alumina crucible. Batches were prepared by mixing CaCO_3_, Al_2_O_3_, and SiO_2_ to form 50 g of 25CaO–20Al_2_O_3_–55SiO_2_ glass (wt %). The glass batches with 0.05 or 0.025 wt % MoO_3_, 0.08 or 0.04 wt % WO_3_, and/or 0.40 wt % carbon were melted and the resultant glass cullet underwent remelting. Note that the MoO_3_ and WO_3_ contents are the same when converted to mol %. After annealing the melt at 850 °C for 30 min, the glass specimen was then heated at 1050 °C for 2 h at a heating rate of 100 °C/h to achieve crystallization. The glass specimens obtained are listed in Table 1. Prior to remelting, some glass specimens were prepared using the same weight additives to the glass cullet. Therefore, the melting of Product-A and -B was conducted under a reductive atmosphere because of the presence of carbon in the glass batch. Product-A corresponds to the same glass specimen reported in our previous studies.^6,13−17^ Product-F corresponds to CAS GC-H prepared by mixing with a glass cullet containing metallic Mo and W particles. For comparison purposes, Product-E was prepared by the reduction of both MoO_3_ and WO_3_. The parent glasses are designated as Parent-X, where X represents the product letters listed in Table 1. To investigate the difference in the oxidation of metal Mo and W particles, the glass batches for Product-C and Product-D also underwent remelting for 2 h to form the glass specimens denoted herein as Parent-C-2 h and Parent-D-2 h. The effect of MoO_3_ on the crystallization behavior was examined by performing heat treatment of the glass with 25CaO–20Al_2_O_3_–55SiO_2_ glass (wt %) with 0.05 wt % MoO_3_ at 1050 °C for 2 h at a heating rate of 100 °C/h.

**Table 1:** List of Product Names in the Present Study

Product names	used MoO_3_ or WO_3_	additives in the remelting process
Product-A	0.05 wt % MoO_3_	same compositional batch
Product-B	0.08 wt % WO_3_	same compositional batch
Product-C	0.05 wt % MoO_3_	
Product-D	0.08 wt % WO_3_	
Product-E	0.025 wt % MoO_3_ and 0.04 wt % WO_3_	
Product-F	0.05 wt % MoO_3_	glass cullet prepared using 0.08 wt % WO_3_

The microstructures and crystalline phases of the products were characterized using scanning electron microscopy (SEM; TM-3000, Hitachi) and powder X-ray diffraction (XRD; XRD-6100, Shimadzu). The volume fractions of the glass specimens after crystallization were approximately estimated using binarized SEM images. Based on previous reports,^14−16^ the SEM images of house-of-cards structures, which are composed of hexagonal platy particles of metastable CaAl_2_Si_2_O_8_, appear as black regions with needle-like particles that correspond to an arbitrary cross section of platy particles in a house-of-cards structure.^15^ For convenience, we tentatively denote the needle-like particles as crystal particles and define their sizes as the longitudinal length. The number of particles in an area of 25,000 μm^2^ was also counted and averaged. The presence of metal Mo and W particles was investigated using transmission spectroscopy (V670, JASCO; equipped with an absolute reflectance measurement unit [ARSN-733, JASCO)]. The profiles in the wavelength ranges of 420–460 and 670–690 nm were instrumentally derived. 1-mm-thick glass specimens were used for the spectroscopic analyses. The darker region (to be described later) of Parent-D-2 h was used for the transmission spectroscopy measurements. The mechanical properties of the glass specimens after crystallization were briefly evaluated by Vickers hardness tests (HMVG20, Shimadzu) with a 1kgf load and a 15 s holding time. Vickers hardness values were estimated using the average of at least fifteen indentation measurements. Prior to measurements, the glass specimens were cut and polished to form appropriately sized or shaped specimens for each analysis method. For the glass specimens after crystallization, the surface layers were removed by polishing.

## Results and Discussion

### Formation of CAS GC Having House-of-Cards Structures with Different Crystal Sizes

**Figure 1:**
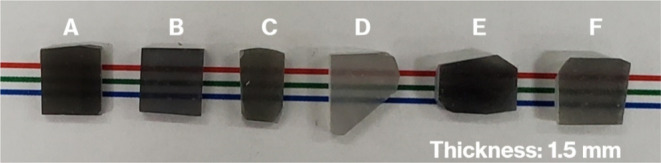
Photographs of Product-A to Product-F.

Figure 1 shows photographs of Product-A to Product-F. The CAS GC-H specimens appeared black due to the presence of metallic Mo and W particles; the glass specimens with metallic W particles were lighter in color than those with metallic Mo particles, which is consistent with previous reports.^14,17^ The difference in color between Product-A and Product-C was minute, in contrast to that between Product-B and Product-D. The colors of Product-E and Product-F were darker than that of Product-D, and Product-E was the darkest. The CAS glass containing 0.05 wt % MoO_3_ after heat treatment at 1050 °C for 2 h was colorless. The XRD pattern for this specimen showed no reflections (data not shown). The 0.05 wt % content of MoO_3_ in the present study is much smaller than those of glass specimens that exhibited crystallization of molybdates via MoO_3_ phase separation.^20,21^ Therefore, the 0.05 wt % content MoO_3_ is not considered to affect the crystallization behavior in the present study.

**Figure 2:**
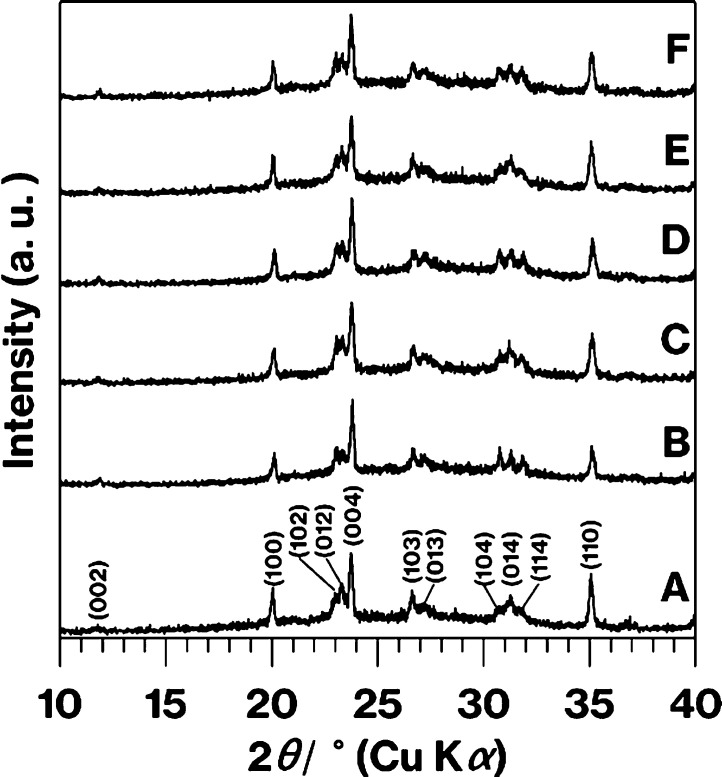
XRD patterns for Product-A to Product-F.

Figure 2 shows XRD patterns for each of the products. The pattern for Product-A matches well with those reported in previous studies.^13−17,22^ All the diffraction lines in the pattern for Product-A are attributed to hexagonal CaAl_2_Si_2_O_8_ crystals, a layered crystal in which the layers are stacked in the *c*-axis direction.^22^ The patterns for Product-B, -C, -D, -E, and -F were similar to that for Product-A. Compared to the pattern for Product-A, the intensity of the (004) reflection^22^ was most pronounced for Product-B. As noted in our previous study,^23^ layered crystals are composed of stacked inorganic layers, of which the crystallinity, stacking order, number, and lateral size have a significant effect on largely affecting the intensity of reflections due to the stacking direction relative to that due to the lateral atom arrangement.^24−27^

**Figure 3:**
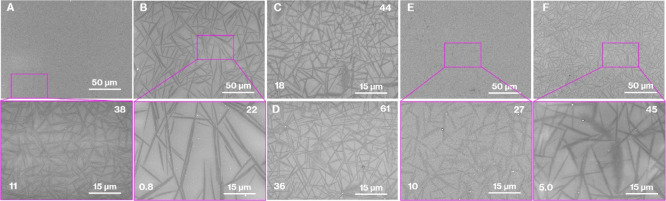
SEM images of Product-A to -F. The number of particles per 225 μm^2^ and the volume fractions (vol %) for Product-A to -F are denoted in the lower-left and upper-right corners of the SEM images, respectively.

**Figure 4:**
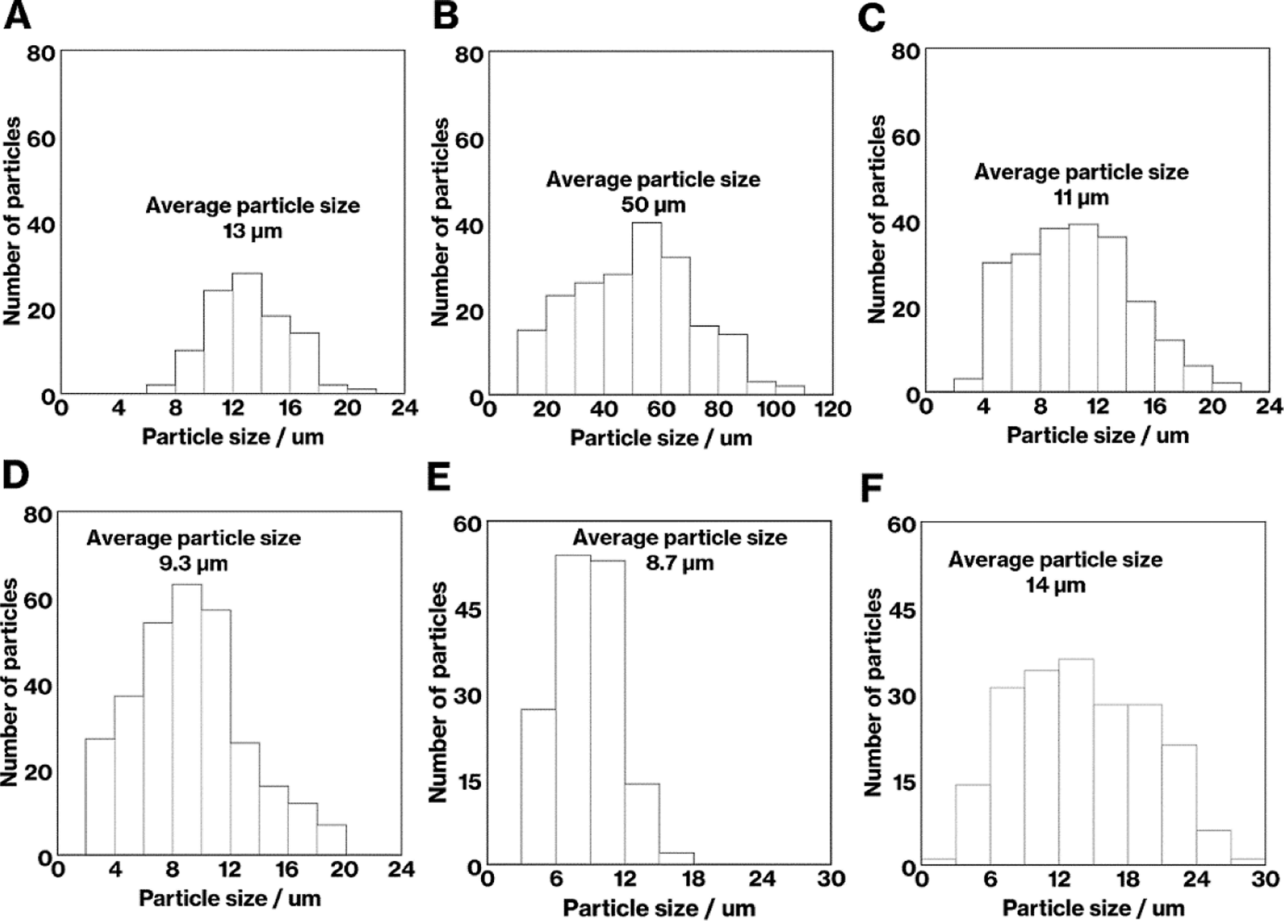
Particle size distributions for Product-A to -F.

Figure 3 shows SEM images of Product-A to Product-F along with the number of crystals and the volume fractions. Needle-like particles were observed in all the higher magnification SEM images (15 μm scale bars). The image of Product-A is consistent with those reported previously.^6,14−17^Figure 4 shows the particle size distributions for Product-A to Product-F. According to Figures 3 and 4, compared to Product-A, Product-B shows an increase in the average size of the needle-like particles from 13 to 50 μm with a decrease in the volume fraction from 38 to 22 vol %. The SEM images of Product-C, -D, and -E appear similar to that of Product-A, while the volume fractions and particle size distributions differ as follows. Compared to Product-A, the volume fraction of Product-C and -D increased from 38 to 44 and 61 vol % with a decrease in the average particle size from 13 to 11 and 9.3 μm, respectively. In addition, the particle size of Product-C and -D are more widely distributed than that of Product-A. Compared to Product-A, the volume fraction of Product-E decreased from 38 to 27 vol % with a decrease in the average particle size from 13 to 8.7 μm. Compared to Product-E, the average crystal sizes in Product-F increased from 8.7 to 14 μm with an increase in the volume fraction from 27 to 45 vol %. The average crystal sizes of Product-F and -A were similar, while the distribution of particle sizes of Product-F was wider than that of Product-A. It should be noted that this study was limited to estimating the aspect ratio and distribution^28,29^ due to the presence of diagonal cross-sections of play particles in an arbitrary cross-section of the house-of-cards structure.

**Figure 5:**
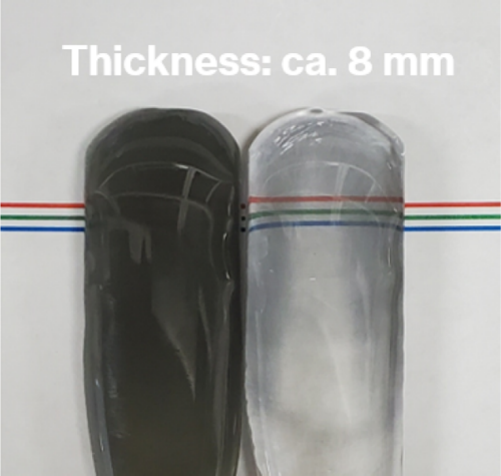
Photograph of Parent-C-2 h (left) and Parent-D-2 h (right).

**Figure 6:**
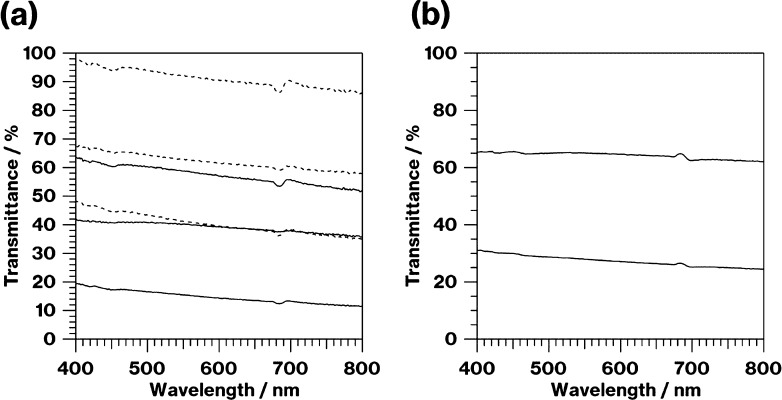
Transmission spectra of (a) Parent-A, -C, -C-2 h (bottom to top; solid lines), -B, -D, and -D-2 h (bottom to top; dashed lines) and (b) Parent-E and -F (bottom to top).

Figure 5 shows photographs of Parent-C-2 h and Parent-D-2 h; the former was darker than the latter and had an uneven color. Figure 6a shows the transmission spectra for the parent glasses. The glasses prepared under an oxidative atmosphere were more translucent than those prepared under a reductive atmosphere. In addition, the translucency of the glasses increased with the melting time under oxidative conditions. Figure 6b shows transmission spectra for Parent-E and -F. The transparency of Parent-F was higher than that of Parent-E. The glass color can have a significant effect on the transparency of the glass specimens; the transparency of Product-D was greater than that of Product-B (data not shown), although the number of crystal particles for Product-D was larger than that of Product-B (Figure 3). The number of glass–crystal interfaces that scatter light is generally proportional to the number of particles.

**Figure 7:**
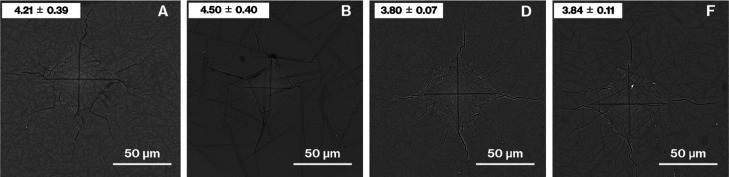
SEM images of Product-A, -B, -D, and -F after the Vickers indentation test. The Vickers hardness (GPa) is denoted in the upper-left corner of each SEM image.

Figure 7 shows SEM images of Product-A, -B, -D, and -E after the Vickers indentation test. All the crack deflections observed in the images of Product-B, -C, and -D were similar to those observed for Product-A. In addition, the crack behavior for Product-A was similar to that reported previously.^14−16^ In contrast, the number of crack deflections for Product-B was clearly less than those for the other glass specimens. The Vickers hardness of the products decreased in the order of Product-B > -A > -F > -D.

Collectively, the SEM images (Figures 3 and 7) before and after the Vickers indentation test and the XRD patterns (Figure 2) reveal the formation of CAS GCs precipitated with hexagonal platy particles of metastable CaAl_2_Si_2_O_8_ crystals that form house-of-cards structures in the present products, which is consistent with previously reported results.^13−19^ The crystal particles in the microstructure were controlled in the range of 2–110 μm (Figures 3 and 4). In our previous study, the sizes of the hexagonal platy particles of metastable CaAl_2_Si_2_O_8_ present in CAS-GC-H with varied compositions and microstructures were in the range of 5–25 μm.^16^ In addition, a decrease in the fracture toughness of CAS-GC-H samples with an increase in the number of particles was discussed, although these glass specimens possessed different microstructures with different compositions.^16^ Therefore, to show the advantage of the present synthesis method, Vickers hardness tests were conducted as a representative evaluation of the mechanical properties of the products (Figures 3 and 7). Given the volume fractions, particle size distributions, and number of particles (Figures 3 and 4), the mechanical properties of CAS-GC-H are likely improved with crystals having both a relatively wide particle size distribution and large volume fraction (Figures 3, 4, and 7). In addition, the crack deflections due to the house-of-cards structure proposed in our previous study^16^ are observed for crystal particle sizes in the 2–110 μm range (Figures 4 and 7). These observations are first revealed by CAS-GC-Hs with varied crystal sizes and one type of microstructure are obtained from the parent glass having one chemical composition. Further study will be required to determine the detailed mechanical properties of CAS-GC-Hs, including those with smaller crystal sizes and narrow size distributions, and larger crystal sizes with an increase in the volume fraction, where the introduction of indentations with varied size, shape,^30^ and load -is necessary based on the error of the Vickers hardness of Product-B, which was larger than those of Product-A, -D, and -F (Figure 7). In addition, the subsurface cracks of CAS GC-H induced by Vickers indentation were recently analyzed using X-ray multiscale tomography.^6,31^ The combination of these two- and three-dimensional analyses could therefore facilitate the elucidation of the mechanisms of the fracture behavior of the CAS GC-H specimens in detail. However, in this study, some of the glass colors (Figure 1) and the crystal sizes (Figure 3) for the CAS GC-H specimens were different; therefore, possible explanations for these results are given in the following subsection.

### Possibility of Effects of Oxidation and Mixing of Nucleation Agents

The free energy for the oxidation of metallic Mo is higher than that of metallic W, according to a previous report.^16^ Therefore, metallic W particles are more easily oxidized than metallic Mo particles, which supports the present results; the colors of the parent glasses containing metallic W particles are lighter than those containing metal Mo particles (Figures 1, 5, and 6). In particular, Parent-D-2 h with uneven coloring contains colorless parts (Figure 5), which suggests the partial disappearance of metallic W particles by oxidation. This may start from the upper part of the glass melt in the crucible. In a previous study,^18^ the microstructures of MAS GCs containing metallic W particles were coarser than those containing metallic Mo particles.^18^ SEM–energy-dispersive X-ray mapping^18^ analysis of the products indicated that metallic W particles were larger than metallic Mo particles. It is also considered that smaller nucleation agents require higher energy for crystallization, which retards crystal growth.^18^ The crystal sizes for the MAS GC specimens containing metallic Mo particles were not significantly dependent on the amounts of MoO_3_ and carbon that were added to the glass batches.^19^ The number of crystal particles was generally proportional to the amount of nucleation agent used.^32^

Based on the larger layered crystals of Product-B grown in the stacking direction with a decrease in the volume fraction, in contrast to Product-A (Figures 2–4), possible reasons for the difference between Product-A and -B are as follows: (1) the sizes of metallic W particles are larger than those of metallic Mo particles and (2) the number of metallic W particles is less than that of metallic Mo particles. The increase in particle size with the decrease in volume fraction from Product-A to -B indicates a decrease in the number of interfaces between crystalline and glassy phases (Figures 3 and 4). Given the transparency of Parent-A and -B (Figure 6a), the increase in the transparency of Product-B from that of Product-A is reasonable (Figure 1).

According to the differences in the color of the glasses between Product-A and -C, and between Product-B and -D as well as the transparency of these parent glasses (Figures 1 and 6), metallic Mo and W particles remain after remelting under an oxidative atmosphere that likely decreases the sizes of the Mo and W particles. Given the decrease in crystal sizes from Product-A to -C and Product-B to -D (Figures 3 and 4), oxidative melting decreases the crystal sizes, and reductive melting induces the growth of crystals. A possible mechanism is as follows: (1) oxidative melting decreases the size of metallic particles, (2) reductive melting causes the growth of metallic particles in the glass cullet where oxides were added as a source of metallic particles, and (3) the second reason also results in a decrease in the ratio of the number of metallic particles to the amount of the parent glass due to the increase in the glass amount by the addition of the same compositional batch (see Table 1). Among these reasons, metallic W particles are strongly affected by oxidation compared to metallic Mo particles, according to the decrease in transparency of the parent glass with an increase in the melting time (Figure 6a) and the significant decrease in the particle sizes of Product-D to -B in contrast to Product-C to -A (Figures 3 and 4), which is due to the free energy for the oxidation of metallic Mo and W particles.^18^ Meanwhile, the oxidation of metallic W particles is utilized for the reduction of metallic Mo particles. Therefore, such a reduction process is feasible in the glass melting stage of Product-F. As a result, metallic Mo particles in Product-F are likely to be larger than those in Product-C, which results in an increase in the crystal sizes of Product-F from Product-C (Figures 3 and 4). Some of the oxidized metallic W particles may also be present in Product-F because the crystal sizes were similar to those observed in Product-D or Product-E (Figure 4). Given the decrease in the number of crystals in Product-E to -F (Figure 3) and the high transparency of Parent-F in contrast to Parent-E (Figure 6b), smaller metallic W particles that do not significantly contribute to crystallization are likely present. In addition, fully oxidized metallic W particles may also occur. An increase in the volume fractions from Product-E to -F (Figure 3) most likely results from the presence of both smaller and larger particles in Product-F than in Product-E (Figures 3 and 4). It should be noted that the microstructure of Product-F cannot be obtained by the addition of WO_3_ and MoO_3_ to the glass batch and their reduction by carbon is based on the microstructure of Product-E (Figure 3). However, the limitation of this study lies in the elucidation of the relationships between the number/size of crystal particles and the parent glass color, because these relationships observed between Product-E and Product-F do not match well with the cases of Product-A and -C or Product-B and -D; a decrease in the number or size of metallic particles are currently possible parameters to increase the transparency of the glass specimens rather than the size, distribution, and volume fraction of crystal particles. Although the GC specimens with dark coloration could find application in the fields of light shielding,^33,34^ interior decorations,^35^ and exterior designs, glass melting under a weak reductive atmosphere that may generate glass specimens with the coloration of MoO_3_ and WO_3_ clusters^36^ is thus worthy of further investigation, and such studies are ongoing in our laboratory.

## Conclusions

We have successfully prepared CAS GC-H products with one microstructure with different crystal sizes in the range of 2–110 μm with the parent glass having one composition by oxidation and/or mixing with metallic W and Mo particles as nucleation agents. The specific microstructures that exhibit improved mechanical properties cannot be obtained without these synthetic methods. The present method could also be utilized to control the microstructure of other GC systems.^3−10^
